# Selecting and testing cultural symbols for character IP: evidence from Mount Tai, China

**DOI:** 10.3389/fpsyg.2026.1946141

**Published:** 2026-09-03

**Authors:** Yu Wang, Changqiang Si

**Affiliations:** 1 Cuilian Business School, Taishan College of Science and Technology, Tai’an, China; 2 School of Arts, Shanghai University of Sport, Shanghai, China

**Keywords:** cultural identity, emotional compensation, EWM-TOPSIS, IP, Mount Tai, SEM

## Abstract

**Introduction:**

In the context of cultural digitalization, how to translate grand cultural heritage into character IP (a proprietary character circulated across media and merchandise, e.g., Kumamon, Labubu) with communicative power and emotional appeal is an important issue for enhancing cultural identity and cultural tourism transformation.

**Methods:**

Taking the cultural symbols of Mount Tai as an example, this study constructs an entropy weight-TOPSIS (EWM-TOPSIS) evaluation model and a structural equation model (SEM) to conduct an empirical analysis from two levels: symbol selection and audience response

**Results:**

We finds that anthropomorphic expression and emotional compensation mechanisms significantly enhance audience emotional resonance and further strengthen cultural identity, consumption, and dissemination intentions. The results show that the effective dissemination of cultural IPs does not rely on the direct presentation of grand narratives, but rather on achieving emotional connection through visual dimensionality reduction and personalized expression.

**Discussion:**

This study provides empirical support for the digital translation of traditional cultural resources and the optimization of new media dissemination mechanisms.

## 1 Introduction

As an ancient civilization with long history, China has nurtured a vast and rich cultural heritage. Today, the digital wave is sweeping the globe, how to bring these ancient symbols dormant in the physical world, closer to the people, just as they have played in the distant past, has become the core of the national cultural digitalization strategy (Bi and Wang, 2021). Creating cultural digital IPs is one of the best bridges connecting traditional culture and modern life. Against this backdrop, Mount Tai, the first UNESCO World Heritage Site recognized for both its cultural and natural significance, is a spiritual totem representing the grand narrative of “national prosperity and peace” for the Chinese nation for thousands of years. Its profound cultural heritage and heavy historical and religious significance give it an irreplaceable role in China’s cultural heritage. Therefore, if this extremely grand, sublime, and solemn cultural symbol of Mount Tai can be translated into a digital IP that resonates with contemporary online trends and evokes emotional responses and consumer desires among young people, then this underlying operational logic can help promote other large-scale cultural heritage sites more universally.

Methodologically, we build upon the purely qualitative speculation of traditional humanities disciplines, incorporating operations research, statistics, and institutional economics to conduct concrete empirical studies at three levels.

Our contributions are as follows: First, regarding the selection of cultural symbols, we constructed an initial decision matrix involving multidisciplinary experts, introduced an EWM-TOPSIS hybrid decision-making model, calculated the weights of each evaluation indicator, and assessed and screened the Mount Tai cultural symbol prototype with the greatest potential for modern IP. Second, in verifying the audience’s psychological mechanisms, we constructed a structural equation model (SEM) based on the stimulus-organism-response (SOR) theory. In questionnaire design, we abandoned the traditional discrete Likert scale and adopted a more granular continuous visual analog scale (VAS) to obtain audience perception scores, validating the reliability and validity of latent variables, model fit, and the complex path of cultural identity. Finally, based on empirical data and considering the unique market laws in China, we shifted our focus to key aspects of IP industry operation, combined with successful international experiences, and proposed a comprehensive reform framework, attempting to create a commercial closed loop for traditional cultural IPs, from content co-creation to physical monetization.

In the early 21st century, influenced by the “Digital library activities in Europe” project and the UNESCO initiative (Saracevic, 2004), research focused on the rescue of physical heritage through “digital preservation” technology, with a focus on exploring the application of technologies such as laser scanning and 3D modeling in site restoration (Fiona, 2007). Later, people gradually realized that simply moving museum exhibits online was essentially still a “material-centric” display (Parry, 2007). When faced with digital archives, the audience lacked deep emotional resonance, and this static archiving, apart from attracting professionals, was difficult to establish cultural identity among digital natives (Chang et al., 2026).

The commercial success of public institutions such as the British Museum and the Metropolitan Museum of Art has shifted the focus of research to the “visual IPization and commodification” of cultural heritage. Research has explored how to extract traditional totems and transform them into symbolic assets with exchange value (Hesmondhalgh, 2018). However, this has also been questioned. Alam Bryman used the concept of “Disneyization” to criticize public cultural institutions for oversimplifying and flattening traditional culture in order to cater to the market (Bryman, 2004). Dean MacCannell is concerned that blind IPization without scientific decision-making basis can easily lead to cultural symbols becoming monotonous visual collages (MacCannell, 2013), which will cause people’s aesthetic fatigue and dissolve the “authenticity” and serious cultural and religious connotations of cultural heritage itself.

Faced with the predicament of low commercial conversion rate of public asset IP, recent cutting-edge research has begun to move beyond simple art design and explore the two major fields of empirical psychology and institutional economics. Based on “media equivalence theory”, audiences tend to regard anthropomorphic digital media as real social actors (Reeves and Nass, 1996). Nicholas Epley et al. have confirmed that endowing non-living cultural symbols with anthropomorphic characteristics and micro-contrast personalities can effectively meet the social connection needs and emotional compensation of young people (Epley et al., 2007). However, such theories are mostly applied to commercial brands and AI customer service, and SEM quantitative verification of the translation efficiency of grand traditional cultural IP is still rare. Public cultural management departments have fallen into the classic “principal-agent dilemma” in IP operation (Jensen and Meckling, 2000), that is, administrative employees who lack market incentive mechanisms, which inevitably leads to a natural lack of intrinsic motivation to take commercial risks and pursue blockbuster IPs. Subsequently, Henry Jenkins’ “participatory culture” and Alvin Toffler’s “prosumerism” theory were introduced into IP governance (Jenkins and Deuze, 2008; Toffler, 1990).

## 2 Research aim

We try to investigate how traditional cultural symbols can be effectively transformed into digital IPs that enhance public engagement with cultural heritage in the context of digital communication. Just by taking the cultural symbols of Mount Tai as a case study, we seek to identify the characteristics that enable heritage symbols to be adapted into communicative and emotionally appealing digital forms, and to examine the mechanisms through which such adaptations influence audience responses. By integrating an EWM-TOPSIS evaluation model with SEM, the study evaluates both the selection of cultural symbols and the effects of their digital representation on emotional resonance, cultural identity, dissemination intention, and cultural tourism consumption intention. Next we further aim to clarify how visual simplification, anthropomorphic expression, and emotional reinterpretation can facilitate the translation of grand cultural narratives into accessible and engaging digital content. The study contributes empirical evidence to the fields of digital heritage communication and cultural heritage interpretation, while providing practical insights for the sustainable activation and dissemination of traditional cultural resources in the digital era.

## 3 Methods

Our research aims to break through the empirical path of traditional cultural symbol dissemination and explore three aspects, which are the reconstruction of cultural tourism IP from a mathematical dimension and psychological empirical research. The methods we rely on mainly involve three aspects.

### 3.1 Multi-criteria decision making

Shandong, China has a long history and culture. It is one of the earliest birthplaces of Chinese civilization. The Mount Tai cultural IP should also be rooted in this land. We selected well-known Mount Tai cultural symbols to analyze their potential for IP development. At the same time, in order to improve objectivity and reduce reliance on subjective preference in assigning indicator importance, we introduced the EWM-TOPSIS in operations research for quantification. This is a comprehensive evaluation method that combines objective weighting method with multi-objective decision analysis. The weight is determined by calculating the information dispersion of the indicators, which mitigates the possible bias caused by personal preferences in subjective weighting to a certain extent (Jing et al., 2024).

First, an initial decision matrix X for the Mount Tai cultural symbols is constructed and normalized to obtain $P={\left(p_{\mathrm{ij}}\right)}_{m\times n}$ , where m is the number of candidates Mount Tai cultural symbols, n is the evaluation index of each cultural symbol, and $p_{\mathrm{ij}}$ represents the feature weight of the cultural symbol i under the index j . All selected cultural symbols are positive indicators, as shown in Table 1.

**Table 1:** Selection of cultural symbols of Mount Tai.

Number	Mount Tai symbols	Their historical origins and cultural significance
1	Sunrise at Mount Tai	It is one of the four wonders of Mount Tai, characterized by the spectacular sunrise and sea of clouds at the summit at dawn. The sunrise scene typically follows a magnificent transformation from darkness to pale white, pink, and finally golden yellow.
2	Bixia Goddess	An important goddess in Taoism, she originated in the Song Dynasty and was regarded as a compassionate mother goddess who was in charge of childbirth, protected women and children, blessed agriculture, and granted children.
3	Cliff Inscriptions	The important cultural relics in the Mount Tai Scenic Area include more than 800 steeles and more than 1,000 cliff carvings, covering the calligraphic styles of various dynasties since the Qin and Han dynasties. It is a field museum of Chinese calligraphy art.
4	Mount Tai porters	The porters of Mount Tai are a traditional occupational group that has long been engaged in carrying goods up and down the mountain and transporting garbage on their shoulders. With a carrying pole and their tenacious perseverance, they transport daily necessities, building materials and even heavy machinery to the top of the mountain.
5	Dai Temple	Dai Temple is the largest and most complete ancient architectural complex still existing on Mount Tai, and is known as one of the four major ancient architectural complexes in China, along with the Forbidden City, the Confucius Temple, the Confucius Cemetery, and the Mountain Resort. Originally built in the Han Dynasty, it was the main site for emperors of successive dynasties to demonstrate their power and to worship the god of Mount Tai.
6	The Fengshan Ceremony	The Fengshan Ceremony was the highest-level sacrificial ceremony held by ancient Chinese emperors at Mount Tai during times of peace and prosperity or when auspicious omens appeared. Its purpose was to report their achievements to Heaven and Earth and to establish legitimacy and divine authority.
7	Mount Tai Stone Guardian	It is a kind of spiritual stone in Chinese folklore used to ward off evil spirits and protect the home, transformed into a boy to block evil influences. It originated from the ancient worship of spiritual stones, using Mount Tai stones as the carrier, and engraved with the words “Mount Tai Stone Guardian.”
8	Mount Tai Daughter Tea	A prized pan-fired green tea from the Mount Tai Scenic Area. Its distinctive characteristics stem from its high-altitude origin and significant diurnal temperature variation; the tea leaves are plump and robust, capable of withstanding multiple infusions, and possess a unique chestnut-like aroma.
9	Mount Tai Jade	Sourced from the foothills of Mount Tai, this material belongs to the serpentine jade family. It typically exhibits colors ranging from deep ink-green to blackish-brown, and is widely regarded as bearing a striking resemblance to “Ink Jade” (Mo Cui), a variety of jadeite.
10	Mount Tai Shadow Play	The traditional theater of the region surrounding Mount Tai, an intangible cultural heritage, it originated during the Ming Dynasty and is primarily performed using shadow puppets crafted from donkey hide. A single performer is responsible for the manipulation, singing, spoken dialogue, and musical accompaniment.
11	Mount Tai Tofu Feast	Centered around delicate tofu crafted from the spring waters of Mount Tai, this dish harmoniously blends the culinary traditions of the Imperial Court with local folk customs, offering a tender and exquisite texture. Its origins trace back to the vegetarian ceremonial meals of the Han Dynasty.

Subsequently, the objective weights of the respective indicators are calculated using the entropy weight method; the information entropy $e_{j}$ for each evaluation indicator is calculated as shown in Equation 1:

$$e_{j}=-\frac{1}{\ln(m)}\sum_{i=1}^{m}p_{\mathrm{ij}}\ln(p_{\mathrm{ij}})$$ (1)

Furthermore, the objective weights $w_{j}$ for each evaluation indicator are calculated using Equation 2, forming the objective weight matrix $W={\left(w_{j}\right)}_{1\times n}$ :

$$w_{j}=\frac{1-e_{j}}{\sum_{j=1}^{n}(1-e_{j})}$$ (2)

W represents the weight of each evaluation indicator j relative to all other indicators, thus, by multiplying $w_{j}$ with the corresponding indicator column j in the standardized matrix $P={\left(p_{\mathrm{ij}}\right)}_{m\times n}$ , we obtain the weighted normalized matrix $Z={\left(z_{\mathrm{ij}}\right)}_{m\times n}$ shown in Equation 3, which represents the actual score of cultural symbol i under the indicator j :

$$\begin{array}{c} Z=P\cdot \mathrm{diag}(W) \end{array}$$ (3)

Based on this foundation, the TOPSIS algorithm is employed to extract, for each evaluation indicator, its maximum and minimum values from the matrix $Z={\left(z_{\mathrm{ij}}\right)}_{m\times n}$ , thereby establishing the positive ideal solution and the negative ideal solution. By calculating the Euclidean distances denoted as $D_{i}^{+}$ and $D_{i}^{-}$ between each cultural symbol and these positive and negative ideal solutions, the relative closeness $C_{i}$ is ultimately derived; this value reflects the comprehensive transformation potential of each symbol, as shown in Equation 4. Finally, the core cultural symbols of Mount Tai are quantitatively ranked based on the numerical values of $C_{i}$ .

$$\begin{array}{c} C_{i}=\frac{D_{i}^{-}}{D_{i}^{+}+D_{i}^{-}} \end{array}$$ (4)

### 3.2 Multi-case comparison

We do not confine itself to the concrete physical design of specific IP characters; rather, it dissects the underlying logic of the cross-media translation of cultural symbols. It employs a comparative multi-case analysis, utilizing viral native internet IPs, such as, Duckyo, Chonky Shiba, and Duckboo as points of reference. Drawing upon visual semiotics, the research deeply decodes the successful paradigms of these youth subcultural symbols across dimensions such as visual flattening, emotional compensation, and grassroots-oriented identity.

Drawing upon case studies that have garnered widespread acclaim in the market in recent years, we explore the feasibility of modernizing and deconstructing the inherent sublime attributes of Mount Tai culture against the backdrop of grand narratives. Through the deconstruction and reshaping of cultural symbols, we analyze how the core connotations of Mount Tai culture identified in Section 3.1 can be seamlessly integrated with “IP Characters”. Furthermore, we demonstrate how, operating within a mechanism characterized by “gap and personification”, these cultural symbols can transcend their traditional, solemn contexts to become organically embedded within new media channels, such as short-form videos, cultural tourism promotions, and policy interpretations, thereby achieving a paradigm shift from being mere cultural totems to serving as “social currency”.

### 3.3 VAS and SEM

To assess the actual communicative efficacy of strategies for the modern translation of Mount Tai cultural symbols within a new media environment, this study draws upon the Stimulus-Organism-Response (SOR) model to elucidate the process by which audience attitudes undergo transformation (Mehrabian and Russell, 1974). Employing purposive sampling, the study selected currently enrolled university students as its subjects, given their status as core consumers of online cultural products. Regarding measurement methodology, the research prioritized a theoretical inquiry into cultural symbol translation strategies, rather than a specific empirical evaluation of a particular IP entity and therefore utilized hypothetical scenarios to introduce the stimulus variables. To enhance data continuity and granularity, we departed from the traditional Likert scale in favor of a Visual Analog Scale (VAS); each response option corresponded to an unmarked slider ranging from 0 to 100, enabling the measurement of subjects’ subjective perceptions regarding various IP translation scenarios.

The reliability and validity testing of the questionnaire, as well as the model fitting in this study, were implemented using the Python. The Pingouin library was utilized to calculate McDonald’s coefficient ω serving as an alternative to the traditional Cronbach’s coefficient α , thereby yielding a more robust indicator of internal consistency for continuous variables (Malkewitz et al., 2023). Concurrently, the Semopy library was invoked to construct a Structural Equation Model (SEM); the structural equations for the SEM adopted in this study are presented in Equation 5:

$$\begin{array}{c} \eta =\mathrm{B\eta}+\Gamma \xi +\zeta \end{array}$$ (5)

Specifically, η is defined as an endogenous latent variable, namely, identification with, affection for, and willingness to disseminate the cultural IP, while ξ is defined as an exogenous latent variable, specifically, the perceived entertainingness and contrast-based stimulation of the cultural IP. B and Γ represent the path coefficient matrices, and ζ denotes the residual vector. Utilizing Python to perform maximum likelihood estimation, the coefficients for the cultural symbol translation strategies are determined, thereby quantifying the driving forces that the strategies of “symbolic dimension reduction” and “anthropomorphic translation” exert upon cultural heritage transmission.

## 4 Materials

Prior to applying EWM-TOPSIS, we assembled an evaluation panel comprising seven experts to mitigate the limitations inherent in assessment systems based on a singular perspective. The panel members included university faculty engaged in the research and teaching of Intangible Cultural Heritage, entrepreneurs in the field of traditional Shandong handicrafts, business school professors, digital media practitioners, and cultural tourism promotion specialists. After reviewing the documentation regarding the candidate cultural symbols listed in Table 1, the expert panel assigned a score to each symbol on a 10-point scale, based on eight specific evaluation criteria, including the depth of cultural and historical heritage, regional cultural representativeness, and the extensibility of visual symbolism. A higher score indicates superior performance of the corresponding cultural symbol within that specific dimension. To reduce extremely subjective biases among the experts, the scores were first examined for abnormal ratings and then aggregated using arithmetic averaging. No abnormal ratings required adjustment, ensuring the original expert judgments were retained, ultimately generated the initial decision matrix X presented in Table 2, which served as the input for the subsequent calculations. Kendall’s *w* values ranged from 0.569 to 0.909 across the eight criteria, indicating moderate to strong agreement among the seven experts, and all Kendall’s *w* coefficients were statistically significant (*p* < 0.001).

**Table 2:** Initial decision matrix for Mount Tai cultural symbols (arithmetic mean of expert ratings).

Number	Mount Tai symbols	X1: Depth of historical heritage	X2: Representativeness of regional culture	X3: Visual symbol extensibility	X4: Anthropomorphizing the reshaping of space	X5: Youth subculture fit	X6: Emotional compensation potential	X7: Transmedia narrative adaptability	X8: Feasibility of Derivatives Development
1	Sunrise at Mount Tai	8.0	8.5	8.0	5.5	8.5	7.5	7.0	7.0
2	Bixia Goddess	9.5	9.2	6.5	6.0	6.5	8.5	6.0	6.5
3	Cliff Inscriptions	9.8	9.0	6.0	3.0	4.0	3.5	5.0	6.0
4	Mount Tai porters	7.5	8.8	7.0	8.5	8.2	8.0	7.5	6.8
5	Dai Temple	9.2	8.5	7.0	4.5	5.0	4.0	5.5	7.5
6	The Fengshan Ceremony	9.8	8.5	5.5	3.0	4.0	3.0	5.5	4.5
7	Mount Tai Stone Guardian	8.5	9.5	8.8	9.6	9.2	9.0	9.4	9.5
8	Mount Tai Daughter Tea	6.0	7.5	6.5	4.0	5.5	5.0	6.0	8.5
9	Mount Tai Jade	7.0	7.5	6.5	3.5	5.0	4.5	4.5	9.0
10	Mount Tai Shadow Play	8.0	8.5	8.2	7.5	7.0	6.5	8.0	7.2
11	Mount Tai Tofu Feast	5.5	7.0	5.0	4.5	6.0	6.5	6.5	5.0
Kendall’s W		0.900	0.569	0.669	0.878	0.885	0.909	0.832	0.814

The key to making comparisons in the cultural sphere lies in avoiding working in isolation. By employing semiotics and a comparative analysis of multiple case studies, this research examines the feasibility of translating cultural symbols into modern internet-based IPs. The IPs selected for comparison are highly representative examples of “native” viral sensations born directly from the internet, such as Duckyo, created in April 2017 by designer. Furthermore, in 2022, the brand collaborated with POP MART to launch a series of workplace-themed blind boxes.

The electronic questionnaire was primarily disseminated through on-campus promotional channels within higher education institutions in Tai’an City, as well as via social media platforms. To reduce potential response biases associated with self-reported questionnaires, the introductory page of the survey included a concealed statement regarding the study’s objectives, alongside instructions emphasizing participant anonymity. In total, links to 578 questionnaires were distributed during the survey period; 426 responses were actually received, yielding an initial response rate of 73.7%. The raw data were subsequently cleaned using the Pandas library. To ensure the integrity and robustness of the SEM covariance matrix, the standard deviation of the scores for all observed variables was calculated for each individual questionnaire; questionnaires exhibiting an extremely low standard deviation were deemed invalid. After excluding 95 invalid responses during the data cleaning process, a final sample of 331 valid questionnaires was obtained. Harman’s single-factor test was conducted (Howard et al., 2024), the first factor explained 31.8% of the total variance, below the 50% threshold. Given that the questionnaire comprised 18 observed variables, the number of valid responses exceeded the number of observed variables by a factor of 10 (Bentler and Chou, 1987), thereby satisfying the statistical requirements for Maximum Likelihood Estimation.

## 5 Results

### 5.1 Potential for IP development of Mount Tai cultural symbols

Based on the evaluation framework comprising the eight indicators presented in Table 2, and drawing upon structured scoring provided by an expert panel, a high-dimensional decision matrix was constructed, the entropy weight method was subsequently applied to assign weights to each indicator, with the results presented in Table 3.

**Table 3:** Weightings of evaluation indicators for the IP potential.

Number	Indicators for IP potential	Indicator weights $w_{j}$
X1	Depth of historical heritage	0.0638
X2	Representativeness of regional culture	0.0158
X3	Visual symbol extensibility	0.0525
X4	Anthropomorphizing the reshaping of space	0.3043
X5	Youth subculture fit	0.1492
X6	Emotional compensation potential	0.2344
X7	Transmedia narrative adaptability	0.0885
X8	Feasibility of derivatives development	0.0916

The results of the weighting calculations reveal the underlying logic of IP development in the contemporary digital era. Among all metrics, “X4: Potential for Anthropomorphic Reinvention” and “X6: Emotional Compensation Potential” exhibit the highest weights, followed closely by “X5: Resonance with Youth Subcultures”. Contrary to traditional assumptions and initial hypotheses, factors such as “X1: Depth of Historical Heritage” and “X2: Regional Cultural Representativeness” carry relatively low weights. This indicates that, in the process of cultural transmission and development, elements such as history, religious sanctity, and traditional culture are not the sole core variables determining an IP’s communicative efficacy within the digital sphere; put another way, these factors constitute a relatively small proportion of the elements contributing to an IP’s ultimate success. Rather, the potential for personification and emotional resonance, specifically, the capacity to provide emotional value to modern individuals at a micro level, serves as the critical driving force determining whether an IP can evolve into a “Super IP”.

After obtaining the weights for each evaluation indicator, the TOPSIS was executed to calculate the Euclidean distances, $D_{i}^{+}$ and $D_{i}^{-}$ , between the 11 candidate cultural symbols and the positive and negative ideal solutions, thereby yielding the evaluation index values $C_{i}$ that reflect their potential for IP adaptation, as presented in Table 4.

**Table 4:** The potential for the IPization of Mount Tai symbols.

Number	Mount Tai symbols	$D_{i}^{+}$	$D_{i}^{-}$	$C_{i}$	Rank
7	Mount Tai Stone Guardian	0.0009	0.0424	0.9785	1
4	Mount Tai porters	0.0083	0.0348	0.8074	2
10	Mount Tai Shadow Play	0.0151	0.0276	0.6460	3
2	Bixia Goddess	0.0202	0.0258	0.5605	4
1	Sunrise at Mount Tai	0.0221	0.0232	0.5124	5
11	Mount Tai Tofu Feast	0.0294	0.0154	0.3446	6
8	Mount Tai Daughter Tea	0.0334	0.0107	0.2426	7
5	Dai Temple	0.0333	0.0100	0.2303	8
9	Mount Tai Jade	0.0368	0.0084	0.1855	9
3	Cliff Inscriptions	0.0412	0.0041	0.0906	10
6	The Fengshan Ceremony	0.0422	0.0034	0.0737	11

The results indicate that the potential for IP development within Mount Tai system of cultural symbols exhibits a tiered structure.

The first tier is occupied by Stone Guardian, a figure of a youth originating from ancient folklore. This character not only possesses high regional cultural recognizability (X2) but also offers significant scope for creative manipulation in terms of anthropomorphic reimagining (X4) and cross-media storytelling (X7). Furthermore, the traditional connotations associated with this figure, such as warding off evil spirits and ensuring safety can be reinterpreted in the modern era as subcultural symbols resonating with the youth, reflecting their desire to combat mental burnout and seek a change of fortune, consequently, the figure holds considerable potential for providing emotional catharsis.

The second tier comprises symbols such as the Mount Tai porters and shadow puppetry, while these symbols evoke strong spiritual resonance and possess powerful visual impact, their inherent, fixed forms present significant challenges to their transformation into fully realized IPs with distinct personalities. Consequently, they are better suited to serve as auxiliary elements that underscore the spiritual essence of Chinese culture.

Notably, symbols such as Mount Tai jade, the cliffside stone inscriptions, and the Fengshan Ceremony received extremely low scores. Although they scored highly in terms of historical and cultural depth, their severe lack of potential for anthropomorphic reimagining (X4) resulted in a low overall ranking. This finding argues that grand narratives often require a compelling storytelling framework to stand any chance of going viral among unfamiliar audiences, within the new media ecosystem dominated by short-form videos and live streaming.

### 5.2 Popular IPs

**Figure 1:**
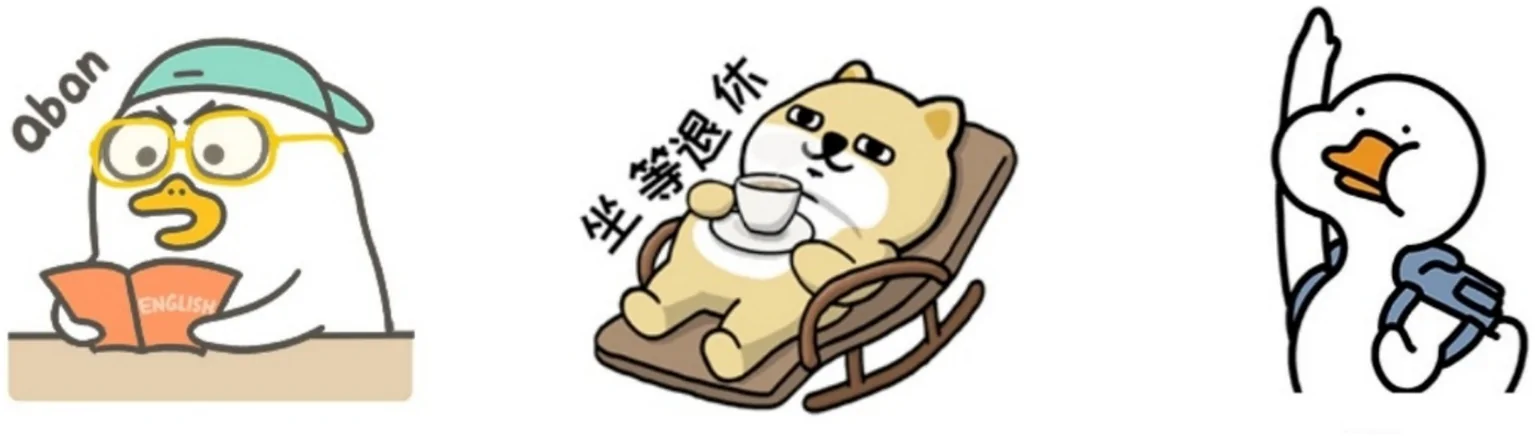
Examples of blockbuster IPs in China (named: Duckyo, Chonky Shiba, Duckboo). Source: Duckyo, Chonky Shiba, Duckboo, WeChat Sticker Platform. Copyright © the original creator. Reproduced for scholarly analysis.

Relying solely on EWM-TOPSIS model to assess the potential of cultural symbols is insufficient to support the development of cultural IPs, therefore, by incorporating a comparative analysis of multiple cases, we conduct an in-depth deconstruction of the underlying logic governing their translation into modern digital media IPs. Representative examples of organically viral IP characters are illustrated in Figure 1.

Traditional cultural IP design often falls into the trap of prioritizing form over essence. To address this, we selected a highly representative, organically viral IP from the contemporary digital landscape as a comparative case study. Employing theory from Roland Barthe of semiotic mythology, we systematically decoded this IP to explore how its creator transformed personal experiences (Barthes, 1964), along with observations of the modern human condition, into a vehicle capable of fostering social consensus, as illustrated in Table 5.

**Table 5:** Semiotic analysis of blockbuster IPs.

IP	Visual features	Psychological connotation	Social features	Success paradigm
Duckyo	Expressive and exaggerated facial features	Fatigue, anxiety, and self-deprecating humor of contemporary youth	A Social Substitute for Workplace Venting and Emotional Release	Discarding refined perfection, embracing authentic imperfection
Chonky Shiba	Rounded physique, a lazy, guileless demeanor appearance	A Longing for warmth and companionship	A social lubricant that mitigates social conflict and offers healing and solace	Providing stress-free digital companionship and a sense of security
Duckboo	Micro-expressions conveying utter existential weariness	The contradictory mindset of yearning to “lie flat” yet feeling utterly helpless	Cyber mouth doubles that subtly refuse and express helplessness	Stripping away grand narratives, focusing on Micro-emotions

### 5.3 SEM

The questionnaire was administered in accordance with the procedures outlined in Section 4. To validate the design of the questionnaire and the effectiveness of the data collection process, we conducted a confirmatory factor analysis based on the 331 valid responses collected, the results of this assessment are presented in Table 6. The standardized factor loadings for all 18 observed variables exceeded 0.70, indicating that the items within the questionnaire possess strong explanatory power regarding their respective latent variables. Regarding internal consistency reliability, we employed the more robust McDonald’s omega coefficient, ω values for all latent variables, as well as their composite reliability scores, were all above 0.80, which exceeding the 0.70 threshold, thereby demonstrating the high reliability of the questionnaire. Furthermore, the average variance extracted for all latent variables exceeded 0.50, confirming that the questionnaire exhibits excellent convergent validity. Discriminant validity was further assessed using the HTMT ratio, with the results presented in Table 7 (Henseler et al., 2015).

**Table 6:** Questionnaire reliability and convergent validity testing.

Variables	Observational items	Standardized factor loadings	MacDonald’s coefficient (ω)	Composite reliability (CR)	Average variance extracted (AVE)
Contrast (S1)	S1-1: Visual novelty	0.842	0.865	0.868	0.687
	S1-2: Breaking stereotypes	0.815			
	S1-3: Approachable & relatable	0.830			
Anthropomorphism (S2)	S2-1: Feels Like a friend	0.822	0.852	0.855	0.663
	S2-2: Engaging than promotion	0.795			
	S2-3: Captures pain points	0.826			
Resonance (O1)	O1-1: Expresses psychological state	0.875	0.891	0.892	0.734
	O1-2: Alleviates psychological resistance	0.852			
	O1-3: Fosters emotional attachment	0.843			
Sense of identity (O2)	O2-1: Perceives the core spirit	0.836	0.870	0.874	0.698
	O2-2: Willing to learn history	0.821			
	O2-3: Endorsement of innovative	0.849			
Willingness to interact (R1)	R1-1: Use exclusive stickers/emojis	0.866	0.885	0.888	0.725
	R1-2: Share short videos	0.845			
	R1-3: Leave comments in livestreams	0.843			
Willingness to buy (R2)	R2-1: Interest in on-site visits	0.812	0.848	0.850	0.654
	R2-2: Purchase cultural products	0.818			
	R2-3: Recommend to others	0.796			

**Table 7:** HTMT ratio.

Variables	Contrast (S1)	Anthropomorphism (S2)	Resonance (O1)	Sense of identity (O2)	Willingness to interact (R1)	Willingness to buy (R2)
Contrast (S1)	–					
Anthropomorphism (S2)	0.742	–				
Resonance (O1)	0.718	0.756	–			
Sense of identity (O2)	0.694	0.731	0.803	–		
Willingness to interact (R1)	0.651	0.688	0.762	0.781	–	
Willingness to buy (R2)	0.623	0.664	0.741	0.768	0.824	–

Maximum likelihood estimation was performed using the Semopy library, in assessing the overall goodness-of-fit of the structural equation model, a selection of both absolute and relative fit indices was employed. The results, presented in Table 8, indicate that the model’s $\chi^{2}/\mathrm{df}$ is 1.076, falling below the critical threshold of 3.0, while the RMSEA is 0.015, which is less than 0.08. Furthermore, the CFI and TLI stand at 0.999 and 0.998 respectively, both exceeding the conventional acceptance threshold of 0.90. Although the model exhibited excellent fit indices, the measurement model in this study was specified *a priori* based on theoretical foundations and previous literature. One possible explanation for the results is the relatively parsimonious measurement model been chosen, comprising six latent constructs with three indicators each, which may have contributed to the excellent model fit.

**Table 8:** SEM fit indices.

Indices	Values
df	129.000
$\chi^{2}$	138.808
CFI	0.999
TLI	0.998
RMSEA	0.015

We further conducted significance tests on the causal relationships within the SEM; detailed data regarding path coefficients β and p -values are presented in Table 9. Result indicates that perceived contrast (S1) exerts a significant positive influence on audience emotional resonance (O1) ( Γ =0.435166, p <0.001). Similarly, anthropomorphic interactivity (S2) demonstrates a powerful positive driving force on emotional resonance (O1) ( Γ =0.575658, p <0.001). These findings validate the prior hypothesis that visual flattening and contextual everyday-ization facilitated through a sense of contrast and anthropomorphic interaction, effectively cater to the psychological compensatory needs of the youth demographic, thereby engendering strong emotional resonance. Furthermore, emotional resonance (O1) serves as an extremely significant positive predictor of audience identification with the IP (O2) ( B =0.828475, p <0.001). This suggests that, within the context of youth subcultures, the process of humorization does not dilute the profound heritage of traditional culture, it fulfills the emotional value sought by contemporary youth, thereby fostering affection and appreciation. Cultural identity holds unique significance for the deepening and dissemination of cultural heritage assets such as Mount Tai. Our data reveal that a sense of cultural identity (O2) significantly boosts the audience’s willingness to engage in interactive dissemination of the IP (R1) ( B =0.863282, p <0.001), as well as their willingness to consume and perpetuate it (R2) ( B =0.666027, p <0.001). This underscores that the true vitality of a cultural IP lies not in static display, but in its transformation through clever symbolism into a fluid social vernacular and a means of self-expression, such as interactive emojis, allowing people to see a reflection of themselves within it.

**Table 9:** SEM path coefficients and hypothesis testing.

Hypotheses	Dependent variables	Path	Independent variables	Coefficient	*p*-value
H1	O1 ( η )	~	S1 ( ξ )	0.435166 ( Γ )	<0.001
H2	O1 ( η )	~	S2 ( ξ )	0.575658 ( Γ )	<0.001
H3	O2 ( η )	~	O1 ( η )	0.828475 ( B )	<0.001
H4	R1 ( η )	~	O2 ( η )	0.863282 ( B )	<0.001
H5	R2 ( η )	~	O2 ( η )	0.666027 ( B )	<0.001

## 6 Discussion

### 6.1 Findings

Through a hybrid empirical approach combining multi-criteria decision-making, semiotic analysis, and structural equation modeling, this study explores the prospects for transforming cultural symbols of Mount Tai into digital IPs. The results of EWM-TOPSIS method indicate that grand narratives, such as the Fengshan Ceremony and historical relics like the stone inscriptions of Mount Tai, while serving as quintessential representatives of Chinese culture, lack the necessary scope for visual simplification and secondary creation, consequently, they prove difficult to directly transform into high-engagement digital IPs. Conversely, through an analysis of organically viral IPs, validated via SEM, we discovered that the everyday reimagining of solemn cultural totems, specifically by imbuing them with relatable micro-expressions, such as those of the working class, above all, emotional resonance relevant to modern life, elicits a profound emotional resonance among the youth demographic ( Γ =0.575658). Furthermore, the data reveals that for IPs rooted in traditional culture, individuals’ sense of cultural identity translates significantly into a willingness to engage via new media platforms ( B =0.863282) as well as a propensity for cultural tourism consumption ( B =0.666027). This suggests that the dissemination of such IPs across digital networks and new media platforms represents a promising avenue for mutually reinforcing traditional culture and the local cultural tourism economy.

### 6.2 Commercialization

Although the audience psychology surrounding Chinese cultural IPs has been explored from a technical standpoint, a broader view of China’s cultural and tourism industry reveals a peculiar cyclical pattern in the creation and operation of state-led public cultural IPs, that they typically begin with administrative directives but end with a cold reception in the market, but the Forbidden City being a notable exception here (Xiao et al., 2025).

The systemic resistance encountered during the transformation of public, state-owned cultural resources into market-oriented cultural capital warrants close attention (Jiang, 2025). Specifically, the rigidity of bureaucratic management mechanisms within the cultural sphere, the imbalance in the distribution of creative proceeds, and the authorities’ excessive vigilance against the potential loss of state assets collectively give rise to a latent principal-agent dilemma within the realm of cultural innovation. By clinging to absolute control over IP assets during their operation, bureaucratic entities become reluctant to delegate authority to grassroots creators, such as street painters and folk artists, which disincentivizes these creators from investing their full effort in turn. An examination of the success trajectories of viral original IPs reveals that their origins lie in the creators’ keen sensitivity to the nuances of daily life, their authentic emotional expression, and the existence of free creative space. Therefore, for bureaucrats, a topic worthy of close attention is how to simultaneously preserve the nominal ownership of cultural resources while thoroughly untying the classic knot so common among institutional employees that whether one works hard or hardly works, the outcome is the same, thereby entrusting the responsibility for IP management to teams that truly possess market acumen.

As evidenced by Japan Production Committee model and the US mature fan-work licensing system (Lerner, 2018), the explosive growth of super IPs often relies on a vast community of secondary creators. The lackluster performance of official IPs in China stems from the absence of a shared mechanism capable of safeguarding the interests of independent creators. In the era of digital natives, in line with the theory of the prosumer, audiences serve not merely as consumers of content, but, more importantly, as its producers (Huntington, 1997). Rather than acting as monopolists of resources, adopting the role of an ecosystem platform builder proves far more conducive to the advancement of cultural creation.

The material foundation for cultural heritage preservation lies in sustained commercial profitability. In contrast to worldwide IPs such as Gundam from Japan or Disney from US (He, 2014), most Chinese official IPs remain stuck at the rudimentary stage of merely selling admission tickets, at best, by the sale of dolls and badges within tourist sites. Moreover, the prices of such cultural and recreational merchandise at these sites are often significantly higher than those of generic souvenirs, the city-themed refrigerator magnets sold by street vendors is an instance.

In this rudimentary stage of physical product sales, there is a distinct lack of high-dimensional monetization strategies for intellectual property. If administrators wish to truly unleash the vitality of traditional cultural IPs, they must transcend mere symbolic representation, instead, eschewing the temptation to avoid complexity. They could systematically overhaul both the governance of public resources and the underlying logic of intellectual property rights, thereby reconstructing a modern commercial ecosystem that spans the entire process from rights confirmation and licensing to revenue sharing.

Unlike other IPs, Mount Tai historically underwrote political legitimacy, it is not an emotionally neutral icon. For instance, the Fengshan Ceremony established the legitimacy of imperial power and an era of the “divine right of kings” (Edelman, 1985; Kertzer, 1988), and it remains a symbol of national memory today. The emotional-compensation framework used in our study lacks integration of these political functions, raising questions that remain to be addressed by future scholars. Anthropomorphic downscaling might reinforce collective identity by making an ancient, sacred symbol more approachable, thereby facilitating its sharing and dissemination among the younger generation. Scholars argue that shared symbols sustain a sense of national affinity, which aligns with Social Identity Theory (Tajfel and Turner, 2004), and the theory of the symbolic construction of community (Cohen, 2013). On the other hand, some critics warn of risks associated with such simplification, may lead to the depoliticization and trivialization of the symbol’s meaning, thereby eroding the historical and political weight it originally carried.

### 6.3 Recommendations

The “IP Dual-Track System” presents a viable option worthy of consideration; it entails a dual-track separation regarding the classification of cultural symbols, strictly distinguishing between the “public service attributes” and the “commercial profit-generating attributes” of the Mount Tai IP. Under this dual-track framework, for commercialized IP ventures, official authorities would co-invest alongside leading market-oriented digital creative agencies to establish hybrid-ownership operating entities. The authorities would contribute their equity stake by valuing and capitalizing the “Mount Tai IP Franchise Rights”, yet would refrain from interfering in day-to-day operations; conversely, the market-oriented team would contribute capital, technology, and operational expertise, thereby holding absolute authority over commercial decision-making (Muehlfeld and Wang, 2022).

We are piloting the launch of the “Mount Tai Digital IP Open-Source Co-creation Library”. This initiative allows independent illustrators, 3D modelers, and short-video creators to access a foundational IP library for derivative creation. Crucially, we are establishing a fair and robust revenue-sharing framework: should these grassroots derivative works generate revenue, whether through market traffic monetization or the sale of physical merchandise, the system will proportionally distribute the earnings between the official licensor and the original creator. Only by safeguarding creators’ intellectual property rights through tangible financial incentives and transparent contractual mechanisms can we foster a virtuous cycle within the entire creative ecosystem, thereby ensuring the continued flourishing and evolution of Mount Tai culture among the younger generation.

## 7 Conclusion

Through a mixed-methods empirical approach, we explore the pathways for transforming traditional cultural symbols into IP. Our findings indicate that, within the context of grand narratives, cultural symbols that lack the potential for visual simplification and secondary creation appear less likely to evolve into blockbuster IPs. Conversely, the everyday reimagining of solemn cultural totems by embracing the emotional value inherent in modern life, elicits a powerful sense of emotional resonance among the youth demographic. Furthermore, data analysis reveals that a strong sense of cultural identity is positively associated with both the willingness to engage in new media interactions and the propensity for cultural and tourism consumption.

Constrained by objective conditions, our research remains subject to certain limitations. Regarding sample selection, our primary focus was on university students; while this demographic constitutes the core consumer base for internet subcultures and digital IPs, this age-specific focus limits the generalizability of our findings across the entire spectrum of audiences. Future research endeavors could therefore be expanded to encompass groups across various age brackets. Furthermore, our causal analysis relied predominantly on hypothetical scenarios embedded within questionnaires and on participants’ self-reported data, thereby limiting the scope of observable audience behaviors.

It also must be acknowledged that, given the use of hypothetical stimuli and the collection of self-reported data over a concentrated period, the structural relationships identified should be interpreted as associations rather than causal effects. The study design does not establish temporal precedence, nor does it fully rule out alternative explanations such as reverse causality or unmeasured confounding factors. Consequently, the path coefficients reflect the strength of theoretical hypotheses within a simulated context, further validation through rigorous experimental design is required before generalizing these findings to actual audience behavior.

Future studies could leverage AIGC technology to develop authentic virtual avatars derived from diverse cultural symbols, enabling the conduct of genuine field experiments and A/B tests on short-video platforms or at physical tourist sites. By employing data mining techniques to capture behavioral metrics, such as actual click-through rates, dwell times, user comments, and purchases of cultural and creative products, researchers could identify key growth points driving the evolution of the industry.

## Data Availability

The original contributions presented in the study are included in the article/supplementary material, further inquiries can be directed to the corresponding author.
